# Editorial: Disabled people exercise physiology: performance and health implications

**DOI:** 10.3389/fphys.2024.1397055

**Published:** 2024-05-16

**Authors:** Javier Yanci, Daniel Castillo, Aitor Iturricastillo, Raúl Reina

**Affiliations:** ^1^ Sports and Physical Exercise Research Group (GIKAFIT), Department of Physical Education and Sport, Faculty of Education and Sport, University of the Basque Country (UPV/EHU), Vitoria-Gasteiz, Spain; ^2^ Valoración del Rendimiento Deportivo, Actividad Física y Salud, y Lesiones Deportivas (REDAFLED), Faculty of Education, University of Valladolid, Soria, Spain; ^3^ Sports Research Centre, Miguel Hernández University, Elche, Spain

**Keywords:** disability, impairment, physical activity, para sport, training, quality of life

According to the World Health Organization (WHO, 2001), disability is a limitation in functionality or bodily structure that results in difficulties carrying out daily tasks and participating fully in society. Globally, approximately 16% of the population, that is, an estimated 1.3 billion people, have some form of disability (WHO, 2023). This number is growing because of an increase in noncommunicable diseases and people living longer (WHO, 2023). While the prevalence of disability may vary depending on various factors such as geographical location, different nations, societal characteristics, culture, age, gender, and many other factors, it is a global reality that affects many people worldwide (Zheng et al., 2022). From a scientific research standpoint, the study of disability is essential. A quick search on the PubMed database yields over 440,000 articles on “disability” term. Similarly, entering “disability” in the Education Resources Information Center (ERIC) produces approximately 50,000 scientific articles. The interest of the global scientific community in people with disabilities seems to be of great significance.

Multiple research studies have described how people with a disability have compromised health, primarily due to the disability itself (Jimenez et al., 2001) and other lifestyle factors (Carty et al., 2021), like different illnesses, co-morbidities, injuries, and associated risk factors specific to the health condition derived from the disability. The health of individuals with disabilities may also be compromised due to fewer opportunities for physical activity and exercise, as well as sedentary habits and lifestyles (Sherrill, 1993). Considering that various studies have shown that people with disabilities have poorer physical or physiological health (Simón-Siles et al., 2022; Townsend et al., 2022) and that both the impact of the impairment itself and lifestyle can significantly affect the physical and physiological characteristics of individuals with disabilities, the study of disabled people’s exercise physiology and its health implications becomes particularly relevant for society today.

Furthermore, engaging in physical activity, physical exercise, or sports has been described as a critical factor in improving health and wellbeing in various populations (Warburton and Bredin, 2019; Psarrou et al., 2023), including people with disabilities (Sweeting et al., 2020). Low levels of physical activity, physical exercise, sports participation, or high levels of sedentary behaviour have become a widespread problem among people with a disability (Carty et al., 2021). There is evidence that people with disabilities have lower levels of physical activity, physical exercise, or sports participation, and spend more time inactive than counterparts without disabilities (Oviedo et al., 2017; Carty et al., 2021; Martin Ginis et al., 2021), which can imply negative consequences for health (Chen et al., 2017). One way to promote physical activity or physical exercise among our population is through various sports modalities. Currently, practising a sports modality, both specific or in an inclusive form, and increasing the participation of athletes who practice it appears to be a relevant platform and reference point for sedentary or non-practising people with a disability.

Similar to research on disability, scientific interest in sports for people with disabilities has become increasingly relevant in recent decades. A search for the term “disability sport” in the PubMed database yields approximately 18,000 articles, of which approximately 15,000 have been published in the last 2 decades. While scientific knowledge about sports for people with disabilities has increased in recent years, and considering that the practice of sports modalities should be done under appropriate health and safety conditions, studying exercise physiology in people with disabilities can be a relevant topic for improving the health and performance of individuals with disabilities.

This Research Topic on “*Disabled People Exercise Physiology: Performance and Health Implications*” is organized for the Frontiers in Physiology community. The main objective of this Research Topic is to analyze sports performance and health, from the physiological perspective, of athletes and people with disabilities. In this Research Topic, seven scientific articles have been published, of which 6 are original articles and 1 article is a systematic review. Forty-one authors from different nationalities have authored the seven articles. Four of the published articles are related to the implications of exercise physiology on the health of people with disabilities. Specifically, the article entitled “*Effects of a 24-week exercise program on anthropometric, body composition, metabolic status, cardiovascular response, and neuromuscular capacity, in individuals with intellectual and developmental disabilities*” aims to investigate the effects of two physical exercise intervention programs on institutionalized individuals with intellectual and developmental disabilities (IDD). The main conclusions of the study were that a low-cost outdoor intervention in contact with nature appears to be more effective for fat mass reduction and that an indoor intervention using weight-training machines appears to be a good method to promote neuromuscular capacity. The objectives of the article entitled “*Steering Does Affect Biophysical Responses in Asynchronous, but Not Synchronous Submaximal Handcycle Ergometry in Able-Bodied Men*” were to evaluate the effects of combining propulsion and steering requirements on synchronous and asynchronous submaximal handcycle ergometry. The authors of the study concluded that asynchronous handcycling or arm ergometry demands a different handcycle technique in terms of torque production and results in higher metabolic responses than synchronous handcycling. The article entitled “*Cardiac Autonomic Modulation Response Before, During, and After Submaximal Exercise in Older Adults With Intellectual Disability*” whose objectives were to describe and compare the cardiac autonomic modulation before, during, and after the 6-min walk test (6MWT) in older adults with and without Intellectual Disability (ID) concluded that the heart rate variability (HRV) in older adults with and without ID is similar during rest, exercise, and recovery and recovery heart rate (HR) kinetics after the 6MWT was slower in older adults with ID. Regarding the systematic review entitled “*Effects of school-based physical activity interventions on physical fitness and cardiometabolic health in children and adolescents with disabilities: a systematic review*,” the objective was to examine the influence of school-based physical exercise programs on physical fitness and cardiometabolic health in children and adolescents with disabilities. This systematic review concluded that school-based physical exercise programs were very efficient in improving health-related physical fitness and skill-related physical fitness in children and adolescents with disabilities, while the evidence concerning the variables of body composition and cardiometabolic health is inconclusive and warrants further investigations. The other three published articles analyze different physical and physiological aspects and their implication on the sports performance of relevant Para sport modalities such as wheelchair basketball and Cerebral Palsy Football. The objectives of the study entitled “*Differences in kinetic characteristics during countermovement jump of football players with cerebral palsy according to impairment profiles*” were to determine and compare kinetic parameters during the performance of a countermovement jump (CMJ) between footballers with cerebral palsy (CP) and non-impaired footballers, and to analyze the differences in this action between different players’ impairment profiles and a group of non-impaired footballers. The results obtained show that the variables related to power production during the concentric phase of the jump are crucial for the performance differences between groups with and without impairment. In the same sports modality, soccer for people with CP, the study entitled “*To What Degree Does Limb Spasticity Affect Motor Performance in Para-Footballers With Cerebral Palsy*?” concluded that the amount of spasticity according to each evaluated joint muscle group of the lower limbs presents a low-to-moderate significant relationship with determined measures of dynamic balance, coordination, horizontal jump, acceleration, and change of direction ability with and without the ball in international-level CP footballers. Finally, a study carried out with wheelchair basketball (WB) players entitled “*Evolution of the internal load and physical condition of wheelchair basketball players during the competitive season*” whose objectives were to describe differentiated perceived training and match load of WB players during the whole season, to analyze the evolution of players’ physical condition changes during a full season and to analyze the association between match load and changes in physical condition during a full season, came to the conclusion that the competitive season represented considerable neuromuscular involvement in these players.

In summary, the Research Topic titled “*Disabled People Exercise Physiology: Performance and Health Implications*,” published in Frontiers in Physiology, provides a novel perspective through various scientific works on physical exercise and its implications for health and sports performance. The information published in the Research Topic can help professionals working in the health and performance field with people with disabilities better understand exercise physiology.
